# Alternatives of mesenchymal stem cell-derived exosomes as potential therapeutic platforms

**DOI:** 10.3389/fbioe.2024.1478517

**Published:** 2024-09-09

**Authors:** Sihun Lee, Se Young Jung, Donghyeon Yoo, Dabin Go, Ji Yeong Park, Jong Min Lee, Wooram Um

**Affiliations:** Department of Biotechnology, College of Fisheries Science, Pukyong National University, Busan, Republic of Korea

**Keywords:** extracellular vesicles, exosomes, nanovesicles, membrane vesicles, regenerative medicine

## Abstract

With outstanding therapeutic potential in the tissue regeneration and anti-inflammation, mesenchymal stem cell-derived exosomes (MSC-EXOs) have emerged as a prominent therapeutic in recent. However, poor production yield and reproducibility have remained as significant challenges of their practical applications. To surmount these challenges, various alternative materials with stem cell-like functions, have been recently investigated, however, there has been no comprehensive analysis in these alternatives so far. Here, we discuss the recent progress of alternatives of MSC-EXOs, including exosomes and exosome-like nanovesicles from various biological sources such as plants, milk, microbes, and body fluids. Moreover, we extensively compare each alternative by summarizing their unique functions and mode of actions to suggest the expected therapeutic target and future directions for developing alternatives for MSC-EXOs.

## 1 Introduction

Exosomes are a group of extracellular vesicles (EVs) that sized to 40–200 nm in diameter that are produced by double invagination of lipid membranes. Notably, it abundantly contains constituents of origin cells (e.g., protein, lipid, mRNA, microRNA (miRNA), surface ligands) since exosomes are derived from invaginated plasma membranes and enriched cargos in endoplasmic reticulum (ER) (Skotland et al., 2017; Kalluri and LeBleu, 2020). Therefore, exosomes can efficiently modulate the cellular function of recipient cells and they play a major role in cell-to-cell communication in various physiological processes including development, and pathogenesis. Furthermore, exosomes feature the genetic pool of the origin cells, they crucially contribute to maintaining tissue homeostasis by toning neighboring cells to be resemble with others. With this regard, there have been numerous efforts to exploit exosomes as cell-free therapeutic nanomedicines for the treatment and diagnosis of various diseases (Samir et al., 2013).

In particular, exosomes from stem cells have emerged as a potential candidate of living stem cell therapy, due to their advantages as non-living materials offer low immunogenicity, high stability, and long-term storage (Choi J. S. et al., 2019; Nagelkerke et al., 2021; Kim H. Y. et al., 2022; You et al., 2022). Among, mesenchymal stem cell-derived exosomes (MSC-EXOs) have been most frequently applied as therapeutics for treating various diseases, such as rheumatoid arthritis (RA) (You et al., 2021a), atherosclerosis (Jayaseelan and Arumugam, 2019), and liver fibrosis (You et al., 2021b). Recently, MSC-EXOs have been applied as anti-aging therapeutics by promoting the migration and proliferation of fibroblasts and stimulating collagen synthesis in the dermis (You et al., 2022).

Nonetheless of their excellent therapeutic potential, clinical application of MSC-EXOs has been limited so far. Particularly, the poor production yield of MSC-EXOs is a significant challenge in the limited production yield of exosomes, due to a finite number of cell division and low exosome biogenesis rate of stem cells. Though there have been many trials in improving exosome production yields, it has remained as a puzzle since adjusting stem cells or their culture conditions can unexpectedly influence the exosomal constituents and functions (Zhu et al., 2017; Cho et al., 2018). Meanwhile, functional variance and low reproducibility of exosomes have also remained as significant hurdles to their practical applications. Since stem cell functions are highly dependent on the physiological environment, exosomal functions vary depending on the donor and its health status, such as age, sex, morbidity of diseases including diabetes, and high blood pressure (Kretlow et al., 2008; Kim et al., 2021). Even, Kretlow et al. (2008) demonstrated that cell passage of stem cells can affect the differentiating potential of exosomes. They reported that as the passage of stem cells increases, the biogenesis of exosomes and their proliferative potential decreases. Additionally, insufficient biological half-life (<6 h) is often indicated as a weakness dragging the therapeutic potential of exosomes. Due to their intrinsic properties, exosomes can be efficiently internalized into cells, and after systemic administration, exosomes tend to accumulate in the liver, spleen, and kidneys rather than reaching their target sites (Lai et al., 2014; Wiklander et al., 2015; You et al., 2021a). To surmount the poor biodistribution of exosomes, several strategies have recently been reported, such as manipulating exosomal surface or adding targeting moiety (Smyth et al., 2014; Tian et al., 2014). However, leakage or denaturation of exosomal cargos disturb the practical application of these approaches so far (Richardson and Ejima, 2019). With this regard, there has been unmet needs for a novel alternative to MSC-EXOs that have excellent therapeutic potential and high production yield and reproducibility, intractable in stem cells.

**FIGURE 1:**
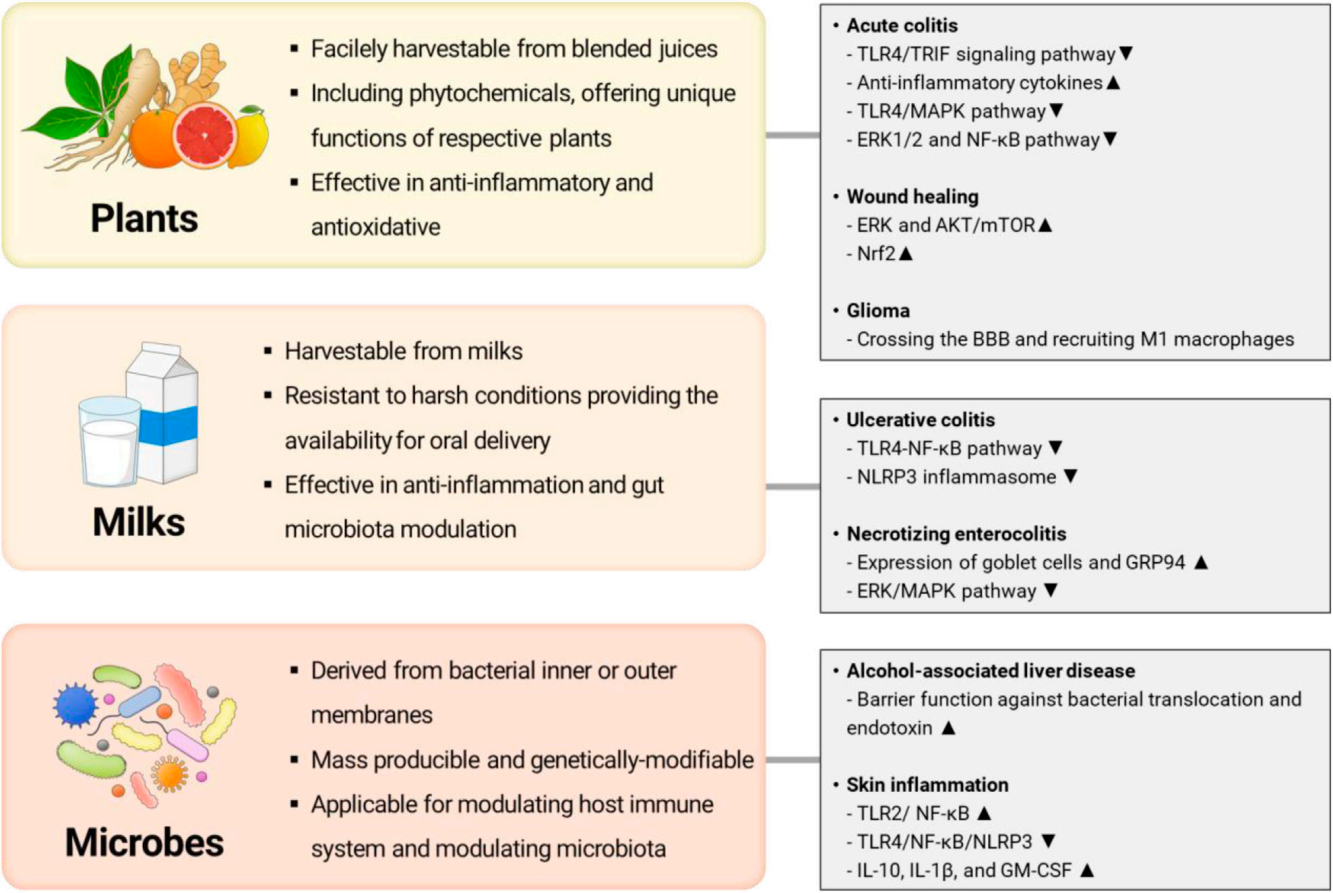
Representative examples of alternative exosomes or exosome-like nanovesicles and their potential therapeutic targets (Illustrated by S. Y. Jung by using Microsoft PowerPoint).

Recently, extracellular vesicles or exosome-like nanovesicles from various biological sources have been suggested as an alternative to MSC-EXOs addressing drawbacks. We illustrated the representative examples of alternatives of MSC-EXOs and their potential therapeutic targets in Figure 1. For example, several researchers have focused on exploiting exosome-like nanovesicles from edible plants (e.g., ginger, lemon, ginseng, and fruit) as novel candidates having potential therapeutic potentials such as anti-inflammatory, antioxidant, anti-cancer, and anti-aging (Raimondo et al., 2015; Teng et al., 2018; Kim and Rhee, 2021; Choi et al., 2024). Otherwise, milk-derived exosomes have recently emerged as potential therapeutics to alleviate arthritis, colitis, and intestinal damage, owing to their excellent anti-inflammatory effects (Arntz et al., 2015; Miyake et al., 2020; Tong et al., 2021a). Meanwhile, membrane vesicles from probiotic bacteria are actively investigated as exosome-like therapeutics to modulate microbiome and relevant diseases (Rodovalho et al., 2020). However, the literature for systematic evaluation and comparison of their functions and characteristics between exosomes or exosome-like vesicles from various sources is lacked, though these studies achieved significant advances in overcoming drawbacks of MSC-EXOs (Ahn et al., 2022; Karamanidou and Tsouknidas, 2022).

Here, we extensively review recent progress in exosomes and exosome-like vesicles as alternatives of MSC-EXOs by summarizing and comparing their advantages and disadvantages as their biological sources, respectively. Thereafter, we briefly suggest appropriate applications of each candidate for the development of therapeutics-based on exosomes or exosome-like vesicles according to their therapeutic implications by reviewing recent studies shown in Table 1.

**TABLE 1:** Alternative of MSC-EXOs and their expected therapeutic outcomes.

Type of source	Source of exosome	Target disease	Expected therapeutic outcomes	Ref.
Plant	Ginger	Triple-negative breast cancer	• Apoptosis, cell cycle arrest, and anti-metastatic effects in TNBC cells▲	Anusha et al. (2023)
		Inflammation	• NLRP3 inflammasome activation▼	Chen et al. (2019)
		Colitis	• Intestinal barrier function▲ • Gut microbiota modulation	Teng et al. (2018)
		Intestinal inflammation	• Internalizing by the intestine cells • LPS-induced inflammation response ▼	Yin et al. (2022)
		Alcohol-induced liver injury	• Expression of liver detoxifying/antioxidant genes • Alcohol-induced ROS▼	Zhuang et al. (2015)
	Ginseng	Skin aging	• Senescence-associated molecules▼ • Melanogenesis-related proteins▼	Cho et al. (2021)
			• Levels of aging-related genes▼ • Protective effects against skin damage caused by UV and oxidative stress	Choi et al. (2024)
			• Regeneration signal delivery, skin stabilization ▲ • Strengthening skin barrier, preventing water loss	Cho et al. (2022)
		Wound healing	• Cell proliferation and migration▲ • Expression of wound healing-related gene	Yang et al. (2023)
		Glioma	• Blood–brain-barrier penetration▲ • Tumor microenvironment modulation	Kim et al. (2023b)
		Melanoma	• Polarization of M2 to M1▲ • ROS▲→ apoptosis of melanoma cells▲	Cao et al. (2019)
		Colitis	• Long-lasting intestinal retention • colon length and wall thickening▲	Kim et al. (2023a)
		Osteoporosis	• Osteoclast differentiation in bone marrow-derived macrophages (BMMs)▼	Seo et al. (2023)
		Inflammatory bowel disease	• Diversity of the intestinal flora▲ • Stability the intestinal barrier ▲	Yang et al. (2024)
	Citrus fruits	Colorectal cancer	• Cell growth inhibitory effect via the macropinocytosis pathway	Takakura et al. (2022)
		Lung, colon, and leukemia cancer	• Cancer cell proliferation▼ • Specifically reaching tumor site	Raimondo et al. (2015)
		Oxidative stress-mediated disease	• ROS▼→ antioxidant and anti-inflammatory effects on human dermal fibroblasts	Urzi et al. (2023)
		Chronic inflammation	• Pro-inflammatory cytokines and protein ▼ • Anti-inflammatory molecules▲	Raimondo et al. (2022)
		Inflammatory bowel disease	• Inflammatory stimuli▼, tight junction protein▲ → restore a functional barrier	Bruno et al. (2021)
		Bile stress	• Msp protein levels▼ → bile resistance of lactobacilli▲	Lei et al. (2021)
Milk	Bovine milk	Osteoporosis	• Proliferation of osteogenic cells▲ • Longitudinal bone growth▲ • Bone mineral density of the tibia▲	Go et al. (2021)
			• Reversing bone loss • Osteoclast presence▼ • Stiffness▲	Oliveira et al. (2020)
			• Bone mineral density▲ • Bone resorption▼	Yun et al. (2020)
		Colitis	• Internalizing into intestinal cells • TGF-β ▲ → therapeutic and anti-inflammatory effect	Reif et al. (2020)
			• Modulating the gut microbiota • Restoring intestinal impermeability • Replenishing mucin secretion	Benmoussa et al. (2019)
		Ulcerative colitis	• Preventing colon shortening • Intestinal epithelium disruption▼ • Regulating intestinal immune homeostasis	Tong et al. (2021a)
		Modulating microbiome	• Modifying intestinal barrier function and immune regulation	Tong et al. (2020)
			• Altering microbial communities in nonbovine species • Crosstalk between bacterial and animal	Zhou et al. (2019)
		Pigmentation	• Melanin contents, tyrosinase activity▼ • Expression of melanogenesis-related genes▼	Bae and Kim (2021)
		Arthritis	• Delaying the onset of arthritis • Cartilage pathology and bone marrow inflammation ▼ • Anticollagen IgG2a levels▼	Arntz et al. (2015)
	Human milk	NEC	• Improving mucosal injury, inflammation, and mucous production	Miyake et al. (2020)
			• Necrotizing enterocolitis severity▼ • Proliferation and migration of IECs▲	Chen et al. (2021)
			• Inflammation and injury to the intestinal epithelium▼ • Restoring intestinal tight-junction proteins	He et al. (2021)
			• Incidence and severity of NEC▼ • Protecting IECs from injury	Pisano et al. (2020)
		Infant immune regulatory	• Anti-CD3-induced IL-2 and IFN-γ ▼ • T regulatory cell▲	Admyre et al. (2007)
			• Abundant immune-related miRNA • Resistance to harsh conditions→ stable delivery to the infant	Zhou et al. (2012)
	Porcine milk	Intestine inflammation	• Cell inflammation▼ • Protecting the IECs	Xie et al. (2019)
Microbes	*Lactobacillus*	Antimicrobial resistant infections	• Expression of host defense genes▲ • Provide protective effects on hosts	Li et al. (2017)
		Colitis	• Maintaining intestinal cell integrity and tight junction • Intestinal Inflammation▼ • Intestinal barrier function▲	Kang et al. (2020)
		Intestinal inflammation	• More effective modulating the immune system than the bacteria themselves • Proinflammatory cytokine▼ • Cross talk between gut microbiota	Vargoorani et al. (2020)
		Hyperinflammatory skin	• Correcting the imbalance between M1 and M2 macrophages • Anti-inflammatory cytokine and immunomodulatory cytokines▲ • M2 macrophage polarization▲	Kim et al. (2020)
		Skin aging	• Cell proliferation▲ • Wrinkle formation and pigmentation▼	Jo et al. (2022)
		Ulcerative Colitis	• Probiotic function • Pro-inflammatory cytokines▼ • Improved the dysregulation of gut microbiota • Diversity of gut microbiota▲	Hao et al. (2021)
		Alcohol-associated liver disease	• Bacterial translocation and endotoxin release▼ • Reversing hepatic fat accumulation, liver enzyme elevation, inflammation, and apoptotic cell death	Gu et al. (2021)
		Atopic dermatitis	• Preventive effect on skin inflammation • Thickening epidermal▼	Kim et al. (2018)
		Skin aging	• Restoring the TNF-α-induced epidermal malformation, abnormal proliferation of keratinocytes • Dermal collagen synthesis▲	Lee et al. (2023)
		Vaginal infections	• Cellular adhesion of lactobacilli▲, preventing pathogens attachment • Colonization of beneficial species▲ • Vaginal homeostasis▲	Croatti et al. (2022)
		Intestinal inflammation	• Maintaining intestinal immune homeostasis • Anti-inflammatory cytokines▲	Hu et al. (2021a)
		Inflammatory bowel disease	• Maintaining colorectal homeostasis • ER stress activation→ LPS-induced inflammation▼	Choi et al. (2020)
		Ulcerative colitis	• Reshaping the gut microbiota • Altering the metabolism pathways of gut microbiota	Tong et al. (2021b)
		Depressive symptoms	• Improving anhedonia and anxiety▼ • Expression of Bdnf in hippocampal neurons▲	Choi et al. (2019a)
	*Bifidobacterium*	Allergy	• Bounding specifically to mast cells • Apoptosis of mast cells without affecting T-cell immune responses • Alleviating food allergy symptoms (e.g., diarrhea)	Kim et al. (2016)
		Allergy	• Polarization of naïve T cells into functional Treg cells▲ • Restoring the cytokine balance	Lopez et al. (2012)
		Intestinal inflammation	• The gut bacterial immune-modulatory effects on the host▲ • Anti-inflammatory effect	Mandelbaum et al. (2023)
		Intestinal inflammation	• Modulating inflammatory responses • Interaction with the host export through immunomodulatory proteins	Rodovalho et al. (2020)
	*Saccharomyces*	Cell wall remodeling	• Cell wall remodeling in fungal cells • Rescuing the yeast cells from antifungal molecules	Zhao et al. (2019)
		Intestinal inflammation	• Antigen presentation activity of dendritic cells▼ • Delivery of immunomodulatory molecules	Nenciarini et al. (2024)

## 2 Plant-derived exosomes and exosome-like nanovesicles

Plants are the most familiar nutrient sources for human beings, and various plant species have been safely consumed as edibles for a long time. With their high accessibility, plants have been broadly utilized as a source of biologically active compounds, and there have been numerous trials conducted to assess their potential as therapeutic agents. Particularly, phytochemicals, a unique type of compounds present in plants, have been identified as essential molecules through which plants exert their therapeutic effects. Various phytochemicals have been discovered to possess anti-inflammatory, antioxidant, antibacterial, and antimycotic efficacy, highlighting the therapeutic potential of plants so far (Rodriguez-Yoldi, 2021; Wasihun et al., 2023; Adil et al., 2024). Besides, plants have an exceptional advantage in the production yield because they can be cultivated and do not require expensive additives, such as growth factors. Therefore, they frequently used as cost-effective sources of therapeutics to date.

On the other hand, in 2009, Regente et al. (2009) reported the presence of exosome-like nanoparticles in sunflower seeds, suggesting the possibility of plant-derived exosomes as a new therapeutic material that combines the properties of exosomes with the benefits of plant. Commonly, plant-derived exosomes can be isolated from the culture medium of callus, a non-differentiated totipotent cell, which can re-differentiate into the original plants. In similar to MSC-EXOs, the multipotent property of callus provides a variety of bioactive components of plant-derived exosomes, and unique phytochemicals in each plant offer indispensable strengths as anti-inflammatory and anti-oxidative therapeutics (Efferth, 2019; Gupta et al., 2023). In addition, the bioactive ingredients of plant-derived exosomes can be adjusted by controlling culture conditions, such as the application of cellular stresses or secondary metabolites. In this sense, plant-derived exosomes have considered as noteworthy by exosome researchers in the early stage (Kim W. S. et al., 2022).

However, several obstacles have remained in the culturing plant cells to produce plant-derived exosomes. Primarily, the culture of plant cells is time-consuming, because most of plant cells are slowly proliferative and susceptible to fungal infections (Xu et al., 2011; Krasteva et al., 2021). Additionally, they often require de-differentiation toward callus to produce certain phytochemicals (Raks et al., 2018; Bansal et al., 2023; Le et al., 2023). Furthermore, some plant cells can be only cultivated in specific conditions, involved in seasonal or regional factors in their habitat, that is difficult to mimic *in vitro* (Mu et al., 2023). Therefore, recent studies have focused on utilizing nanovesicles in grinded juices from plant sources rather than using exosomes from callus cultured medium.

In similar to plant-derived exosomes, plant-derived exosome-like nanovesicles (PELNs) are known to contain natural bioactive components such as lipids, nucleic acids, proteins, and small secondary metabolites from their distinct plants, which present anti-inflammatory, antioxidant, and anticancer activities (Kim J. et al., 2022). Moreover, PELNs have similar size and morphology to mammalian extracellular vesicles (MEVs) and can be internalized into mammalian cells, which makes PELNs able to deliver their cargo and regulate cellular response (Woith et al., 2019). As PELNs are expected to be internalized into human cells and adjust abnormal cell activities by their anti-inflammatory, antioxidant, and anticancer properties, studies about PELNs have been actively made. Recent efforts have suggested the efficient purification and separation of PELN to evaluate their properties, cargo, and efficacy. PELNs are generally extracted from plant juice or homogenized plants and separated by ultracentrifugation, polymer precipitation, immunoaffinity capture, size-based, and microfluidics-based techniques (Mu et al., 2023). Since plants grown on a large scale in a variety of environments are used in this extraction, and because it does not require expensive culture media, growth factors or strict environmental controls, PENLs can be mass produced and are more cost-efficient (Logozzi et al., 2022; Jang et al., 2023). Therefore, there have been substantial efforts in developing PELNs as potential therapeutics.

Nonetheless, research on PELNs is still in its infancy, and the biological function of PELNs and their uptake at the cellular and molecular level are not fully understood. For instance, the bioactive components in PELNs may cause unknown side effects. Therefore, investigation of pharmacokinetics, pharmacodynamics, safety, stability, and efficacy are required to understand their clinical applications. Additionally, most *in vivo* experiments involving PELNs have focused on oral administration. Therefore, intravenous injection is rarely selected and studies on other administration routes are insufficient. According to the research of Cao *et al.*, distribution and degradation tendencies in mouse models depend on the administration route of PELNs (Cao et al., 2019). However, Chen *et al.* expressed concern that intravenous injection of PELNs may stimulate immune system activation, and cause hepatorenal toxicity and hematologic changes (Chen et al., 2022). With this regard, a more in-depth study of the biological interaction of PELNs through various routes of administration is recommended to carefully evaluate their therapeutic applications and biological safety.

### 2.1 Ginger-derived exosome-like nanovesicles

Ginger-derived exosome-like nanovesicles (GELNs) are one of the most well-known PELNs as a potential immunomodulator, since the excellent anti-inflammatory effect of ginger, featured to 6-gingerol and 6-shogaol (Zhang et al., 2016; Mehrotra et al., 2024). In this respect, Zhuang *et al.* investigated the lipid profile of GELNs and discovered that most of the shogaol in ginger extract is imbibed in GELNs. Furthermore, exosome-like characteristics of GELNs offer enhanced cellular internalization of 6-shogaol and thereby effective nuclear translation of nuclear factor erythroid2-related factor 2 (Nrf2) through the TLR4/TRIF pathway, resulting in protection against inflammatory disorders by inhibiting oxidative stress at the targeted site (Semwal et al., 2015; Zhuang et al., 2015). Therefore, GELNs have extensively studies as potent candidates exerting anti-inflammatory, antioxidant, antimicrobial, and anticancer effects to date.

For example, GELNs exhibit the excellent therapeutic potential in alleviating inflammatory bowel diseases (IBDs) by suppressing intestinal inflammation and reprogramming gut microbiota. In the DSS-colitis mouse model, GELNs show reduction of acute colitis and promotion intestinal repair through the reduction of pro-inflammatory cytokines (TNF-α, IL-6, and IL-1β) and increase of anti-inflammatory cytokines (IL-10 and IL-22) (Mehrotra et al., 2024). Zhang et al. (2016) claimed that the facilitated internalization into IECs and macrophages may contribute to the potent anti-inflammatory effect of GELNs. Additionally, Zhuang *et al.* found that GELNs are preferentially taken up by *Lactobacillus rhamnosus* (LGG) and the miRNAs of GELNs, such as mdo-miR7267-3p, induce LGG to metabolize tryptophan to indole-3-carboxaldehyde (I3A). I3A produced by LGG works as a ligand for aryl hydrocarbon receptor (AHR) and promotes the expression of IL-22, which is critical for the maintenance of intestinal epithelial cells (IECs) (Teng et al., 2018).

### 2.2 Ginseng-derived exosome-like nanovesicles

Along with GELNs, ginseng-derived exosome like nanovesicles (GDNPs) have been considered as one of the most prominent PELNs, providing excellent anti-oxidative and anti-inflammatory capabilities. Ginseng contains a variety of bioactive ingredients, such as polyphenols, and acidic polysaccharides that possess anti-inflammatory potential of ginseng. In particular, ginsenosides, a unique group of phytochemicals of ginseng, have been highlighted as potential therapeutic candidates, due to their antioxidant, anti-cancer, and anti-aging potency. Therefore, GDNPs have been applied in treating inflammatory diseases from the early stages of PELN research. For example, ginsenosides exhibit anti-inflammatory potential in treating inflammatory bowel disease (IBD) by suppressing the Mitogen-activated protein kinase (MAPK)/Nuclear factor kappa-B (NF-κB) pathway (Ahn et al., 2016). Similarly, Yang *et al.* investigated the anti-inflammatory effect of GDNPs in treating IBD by upregulating Nrf2 signaling pathway, which promotes antioxidant activity when they internalized into intestinal cells. Additionally, GDNPs downregulated the inflammation-related TLR4/MAPK signaling pathway and protecting macrophages from LPS induced oxidative damage, like ginsenosides (Yang et al., 2024). Further, Kim *et al.* insisted that GDNPs promote an anti-inflammatory response not only by suppressing the NF-kB pathway and pro-inflammatory cytokines, but by enriching beneficial bacteria, such as bacteroidota, altering the gut microbiota and reinforcing gut homeostasis (Kim et al., 2023a).

At the meantime, the immune regulating effects of GDNPs have been also highlighted in cancer immunotherapy field. In the tumor microenvironment (TME), tumor-associated macrophages (TAMs) are mostly polarized to the M2-phenotype that adjusts TME toward pro-tumorigenic by facilitating the tumor growth, angiogenesis, invasion, and metastasis (Xiao and Yu, 2021). Interestingly, recent studies have revealed that GDNPs have a potential in reprogramming tumor microenvironment (TME) toward anti-tumorigenic by altering tumor-associated macrophages (TAMs) from M2 phenotype to M1 phenotype. For instance, Cao *et al.* demonstrated that GDNPs significantly suppress M2-like polarization and induce M1-like polarization of macrophages through TLR4 and MyD88 signaling pathway. And the polarization of M1-like macrophages promotes secretion of proinflammatory cytokines and oxidative compounds, inducing apoptosis of melanoma cells both *in vivo* and *in vitro* (Cao et al., 2019). In addition, therapeutic approaches using GDNPs for treating glioma have recently been proposed. Glioma is one of the most fatal and challenging type of cancers, derived from glial cells in the brain, and it is mostly intractable since the existence of blood-brain barrier (BBB), blocking the delivery of therapeutics into the brain. However, Kim et al. (2023b) confirmed that GDNPs can efficiently cross the BBB and recruit M1 macrophages into the TME, regulating TAMs and inhibiting glioma progression (Lin et al., 2021).

Increasing attention to the functions and properties of GDNPs has led to additional research. For example, Cho *et al.* confirmed that GDNPs downregulate melanogenesis-related factors (tyrosinase, tyrosinase-related protein 2, and Ras-related protein 27) and upregulate high mobility group box 1 (HMGB1). As a result, GDNPs significantly alleviated UVB stress and apoptosis of melanocytes, and thus represented the anti-senescence and anti-pigmentation effects (Cho et al., 2021). Additionally, Yang *et al.* investigated the wound healing effect of GDNPs in both *in vivo* and *in vitro* models. The study has showed that GDNPs enhance wound healing by activating the extracellular signal-regulated kinase (ERK) and AKT/mechanistic target of rapamycin (mTOR) pathways, which plays a critical role in cell proliferation and growth (Yang et al., 2023).

### 2.3 Citrus fruits-derived exosome-like nanovesicles

Citrus fruits are common source of antioxidants, including vitamins. Due to their abundant micronutrients, such as folic acid, carotenoids, flavonoids, and limonoids, vitamin C, they frequently pointed as a potential source of phytochemical, possessing antioxidant, anti-inflammatory, and anti-cancer properties (Silalahi, 2002; Zou et al., 2016). Furthermore, the intrinsic property of exosome and exosome-like nanovesicles allow the stable transfer of the contents into human cells, citrus fruits-derived exosome-like nanovesicles have been extensively studied as a potential anti-oxidative medicine. For example, citrus limon-derived exosome-like nanovesicles (LDEVs) harbor various citrus originated bioactive micronutrients and they can intracellularly deliver their cargos efficiently. According to the study, reported by Baldini et al. (2018), LDEVs contain detectable amounts of citric acid and vitamin C, and exert an antioxidant response in human cells by delivering their antioxidants. Moreover, Urzi *et al.* demonstrated that LDEVs exhibit the protective effects against cellular oxidative stresses by translocating the AhR into the nucleus, activating Nrf2, and upregulating the transcription of antioxidant enzymes (Urzi et al., 2023).

In the meantime, LDEVs have been investigated as a potential candidate for treating inflammatory diseases. For instance, Raimondo et al. (2022) revealed that LDEVs can alleviate LPS-induced inflammation by inhibiting the extracellular signal-regulated kinase 1/2 (ERK1/2) and NF-κB signaling pathway. With the discovery in the biological implications, LDEVs have been intensively encouraged as further studies in treating IBDs. As an example, the therapeutic potential of LDEVs in IBD was examined by Bruno *et al.* They found that LDEVs can alleviate epithelial barrier inflammation and normalize intestinal permeability in inflammatory cytokine-stimulated colonic epithelial cells. Additionally, they estimated that is up to the upregulation of inflammatory-related genes such as ICAM1 or HMOX-1 and restored tight junction proteins such as claudins and occludin (Bruno et al., 2021). Meanwhile, the gut microbiome is highly associated to the maturation of host immune system and the regulation of intestinal inflammation (Zhao et al., 2023). With this regard, Lei et al. (2021) evaluated the influence of LDEVs on reprograming the gut microbiome. Bile acid is one of the major metabolites that modulate gut microbial composition, and interestingly, LDEVs appears to enhance the bile resistance of *Lactobacillus rhamnosus* GG (LGG). Additionally, they confirmed that LDEVs increase the resistance against bile acids of LGG by downregulating protein expression of Msp1 and Msp3. The results suggest that LDEVs can contribute to alleviate IBDs by increasing the proportion of probiotics strains.

Mounting studies suggests that the therapeutic effects of LDEVs might be attributed to abundant flavonoids in citrus fruits (e.g., eriocitrin, quercetin, naringin, and limonin) (Carullo et al., 2017; He et al., 2020; Song et al., 2021). Thus, several studies have focused on exploiting the function of anti-cancer and antiproliferative effects of citrus flavonoids such as nobiletin, quercetin, taxifolin (Benavente-Garcia and Castillo, 2008). Especially, the anti-proliferative effect of flavonoids compounds in TNF-related apoptosis-inducing ligand (TRAIL) receptor-low expressing or p53-mutated cells make LDEVs attractive anticancer drugs. For example, Raimondo *et al.* reported the anti-proliferative effect of LDEVs in lung, colon, leukemia cancer cells. Furthermore, they suggested the mode of action of LDEVs to inhibit cancer cells, and it is dependent to upregulation of pro-apoptotic mRNAs, TRAIL-receptor expression, and downregulation of cell survival-associated proteins. With this regard, they confirmed that LDEVs can suppress the tumor growth and it was mediated by the reduction of pro-angiogenic cytokines VEGF-A, IL6 and IL8, in addition to TRAIL-mediated apoptosis (Raimondo et al., 2015). Similarly, Takakura et al. (2022) also reported that the contribution of LDEVs in tumor growth inhibition on colorectal cancer (CRC). They suggested the inactivation of p53 plays a key role in suppressing tumor growth by promoting the micropinocytosis and thereby internalization of LDEVs.

### 2.4 Other PELNs

In early 2010s, Ju *et al.* spotlighted the therapeutic potential of grape exosome-like nanoparticles (gELNs) in alleviating intestinal tissue regeneration. They found that gELNs exhibit the targeting ability to intestinal stem cells and restore intestinal structures by promoting dramatic proliferation of intestinal stem cells and robustly accelerating mucosal epithelial regeneration (Perut et al., 2021). From the discovery of gELNs, various PELNs with unique therapeutic effects have been discovered, such as antioxidant, anti-cancer, anti-aging effects.

As an interesting example of antioxidative PELNs, Perut *et al.* reported that PELNs derived from strawberry juice, and these PELNs featured to high anthocyanin, folate, flavonol, vitamin C content, and high antioxidant capacity. With abundant natural antioxidants content, strawberry juice-derived PELNs show preventive effects against oxidative stress in MSC cells (Ju et al., 2013). Likewise, Kim *et al.* demonstrated that carrot-derived EVs (Carex) significantly inhibited ROS production and cell death in myocardial infarction and Parkinson’s disease mimicking *in vitro* model. They also suggested that Carex can reduce the expression of antioxidant molecules, including Nrf-2, HO-1, and NQO-1 (Kim and Rhee, 2021).

Additionally, Trentini et al. (2022) investigated the anti-inflammatory effect of apple-derived nanovesicles (ADNVs) and demonstrated that ADNVs negatively affect the activity of TLR4 and downregulate the NF-κB inflammatory pathway. ADNVs thus can prevent extracellular matrix degradation by increasing collagen synthesis (COL3A1, COL1A2, COL8A1, and COL6A1) and downregulating metal proteinase production (MMP1, MMP8, and MMP9). With this regard, they suggest ADNVs as novel candidate of anti-aging therapeutics and skin aesthetic ingredients (Trentini et al., 2022). Interestingly, Fujita *et al.* applied ADNVs in modulating intestinal permeability. They discovered that several PELNs can exert modulating effects on intestinal functions, and in particular, ADNVs can permeabilize large particles in the intestinal barrier by upregulating the expression of organic anion-transporting polypeptide in epithelial cells.

Meanwhile, EVs derived from callus cultured edelweiss (EDEVs) have been recently reported as a potential skin regenerative medicine. Edelweiss is a plant that has been used in traditional medicine for a long time, since they contain various antioxidant and anti-inflammatory polyphenolic secondary metabolites (Daniela et al., 2012; Marlot et al., 2017). With this nature, Daniela et al. (2012) evaluated therapeutic effects of EDEVs and exhibited EDEVs inhibiting production of ROS and melanin in human skin cells. They suggested the containment of filaggrin (FLG), aquaporin 3 (AQP3), and collagen I (COL1) in EDEVs confer skin strengthening effects. On the other hand, several research groups have focused on anticancer potential of PELNs. For example, Chen *et al.* reported that nanovesicles from tea flowers (TFENs) exhibit anticancer activity by stimulating ROS generation. They discovered that TFENs can induce ROS-mediated mitochondrial damage and thereby cell cycle arrest, and the subsequent cell apoptosis.

### 2.5 Limitations of PELNs

As mentioned above, mounting studies points to PELNs as a novel and attractive treatment for skin diseases, IBD, and cancer. And since PELNs have various advantages, including high biocompatibility and low biotoxicity, cost-effectiveness, in addition to diverse intrinsic therapeutic activity, PELNs are emerging as a promising therapeutic agent to replace MSC-EXOs.

Nonetheless, research on PELNs is still in its infancy, and the biological function of PELNs and their uptake at the cellular and molecular level are not fully understood. For instance, the bioactive components in PELNs may cause unknown side effects. Therefore, investigation of pharmacokinetics, pharmacodynamics, safety, stability, and efficacy are required to understand their clinical applications. Additionally, most *in vivo* experiments involving PELNs have focused on oral administration. Therefore, intravenous injection is rarely selected and studies on other administration routes are insufficient. According to the research of Cao *et al*., distribution and degradation tendencies in mouse models depend on the administration route of PELNs (Cao et al., 2019). However, Chen et al. (2022) expressed concern that intravenous injection of PELNs may stimulate immune system activation, and cause hepatorenal toxicity and hematologic changes. With this regard, a more in-depth study of the biological interaction of PELNs through various routes of administration is recommended to carefully evaluate their therapeutic applications and biological safety.

On the other hand, reproducibility is cited as the biggest obstacle in studying PELNs. Typically, the degree of variation between plants is much greater than that between animals and there are significant differences in particle size, structure, yield, purity, and dispersibility of extracellular vesicles produced from different plant species (Mu et al., 2023). Moreover, to date, there are no standardized protocols or guidelines for the isolation and purification of PELNs. Various methods can be used to purify PELNs. Among them, differential centrifugation and ultracentrifugation are the most commonly used, and various methods such as tangential flow filtration, ultrafiltration, and size exclusion chromatography are also used. Hence, depending on the purification method and the number of iterations, the size, content, and purity of PELNs may differ, resulting in heterogeneity and affecting experimental results (Ly et al., 2023). Overall, insufficient research is the main cause of these limitations. Therefore, advanced studies of PELNs will open up the possibility of future materials that can replace MSC-EXOs.

## 3 Milk-derived exosomes

Breast milk is a natural nourishing liquid containing milk fat globules, immunocytes, and a variety of soluble proteins such as immunoglobulins, cytokines, and antimicrobial peptides, developing the infant immune system and playing a crucial role in infant growth (Armogida et al., 2004). In particular, recent studies reported that the breast milk abundantly contains exosomes and they play roles in the modulation of infant immunity (Admyre et al., 2007), the prevention of necrotizing enterocolitis (NEC) in infants (He et al., 2021), and the composition of the gut microbiota (Jost et al., 2014). Furthermore, breast milk-derived exosomes exhibit an exceptional resistance against harsh conditions to transfer genetic materials to the infant, therefore, it has emerged as an attractive material to deliver miRNA and mRNAs in oral or transdermal delivery (Zhou et al., 2012).

### 3.1 Bovine milk-derived exosomes

Among them, bovine milk is the most frequently and familiarly used source of milk-derived exosomes. In particular, recent studies have highlighted the therapeutic effects of bovine milk-derived exosomes (BMDEs) in inflammatory bowel disease (IBD) by alleviating inflammation, restoring intestinal barrier, and modulating gut microbiota. For example, Tong et al. (2021a) reported that BMDEs alleviated the inflammatory response in dextran sulfate sodium (DSS)-induced ulcerative colitis (UC) mice model. BMDEs restored intestinal cytokine homeostasis and immune cell balance between IL10^+^Foxp3^+^ Treg cells and Th17 cells by inhibiting the TLR4-NF-κB signaling pathway and NLRP3 inflammasome, thereby alleviating mouse UC (Tong et al., 2021a). Additionally, several studies demonstrated the potential of BMDEs in restoring intestinal barrier functions. For instance, Li et al. (2019) presented the new insights of the development of NEC, associated with a reduction in goblet cells and glucose-regulated protein 94 (GRP94) + cells, the potential of BMDEs, preventing NEC by enhancing the expression of goblet cells and GRP94 was suggested. In recent study, to evaluate as prebiotics for human infants, Tong *et al.* observed that BMDEs alter the composition of intestinal microorganisms, regulate short-chain fatty acids (SCFAs), and increase the expression of genes (e.g., Muc2, RegIIIγ, Myd88, and GATA4) important to mucus layer integrity (Tong et al., 2020). Furthermore, BMDEs restored disturbed gut microbiota by enriching beneficial microbiota such as *Akkermansia* (Tong et al., 2021a). Interestingly, Reif *et al.* comparatively evaluated the therapeutic effects of BMDEs and HBM-derived exosomes and the results suggested that BMDEs have a similar anti-inflammatory effects to HBM-derived exosomes in DSS-induced colitis mice (Reif et al., 2020). Meanwhile, milk exosomes have been exploited to treat skin pigmentation or osteoporosis. For example, Bae and Kim (2021) proved that miR-2478 of BMDEs inhibits the expression of Rap1a and reduces melanin production through the Akt-GSK3β signal pathway (Bae and Kim, 2021). Furthermore, Oliveira et al. (2020) show bone modulation potential of BMDE, through bone loss models such as diet-induced obesities and ovariectomy (OVX) mice.

Meanwhile, BMDEs have been highlighted as potential drug delivery carriers for oral delivery. Typically, most biological macromolecules exhibit poor bioavailability in an oral delivery route, due to the enzymatic degradation, denaturation in the gastrointestinal juice, and the intestinal epithelial barrier. Furthermore, absorbed molecules are mostly removed in the liver by the first-pass effect. Therefore, the administration route has been limited to intravenous so far (Thanki et al., 2013; Betker et al., 2019; Han et al., 2024). Interestingly, recent studies revealed that BMDEs have a unique capable for passing through the intestinal epithelial barriers effectively. Therefore, they have been actively applied to deliver protein or gene-based drugs by oral administration (Betker et al., 2019; Han et al., 2022). Specifically, bovine IgG in BMDEs allows the evasion of lysosomal degradation by binding to Fc receptor (FcRn) (Ward et al., 2003; Samuel et al., 2017). As the binding of IgG to FcRn has a high affinity in the acidic environment of the GI tract, this interaction plays a key role in the intestinal absorption capacity of BMDEs (Martins et al., 2016; Betker et al., 2019). With this regard, BMDEs have emerged as promising platform that enables the oral administration of various therapeutic agents in the pharmacological field (Benmoussa et al., 2016; Agrawal et al., 2017; Zempleni et al., 2017; Han et al., 2022).

### 3.2 Other milk-derived exosomes

In the meantime, milk-derived exosomes from other mammalian, such as pigs, or sheep milk-derived exosomes were evaluated as potential candidates. For example, Xie et al. (2019) found that miR-4334 and miR-219 of porcine milk exosome reduced LPS-induced inflammation and cell death through the NF-kB pathway and miR-338 through the p53 pathway. In addition, comparison between exosomes derived from bovine milk and sheep milk was made by Quan et al. (2020), demonstrating that miR-26a, miR-191, let-7f, let-7b, and miR-10b have significant effects on neonatal growth and development.

On the other hand, the assessment in human breast milk-derived exosomes (HBM-EXOs) relatively remained in the early stage, since their uses are limited by issues in ethics and reproducibility. However, mounting evidence suggests that HBM-EXOs play a crucial role in developing innate immunity of infants by strengthening gastrointestinal barrier functions and modulating gut microbiota. For example, Chen et al. (2021) have been demonstrated that HBM-EXOs contribute to protect infants against NEC, a leading cause of neonatal mortality, by mitigating the lipopolysaccharide (LPS)-induced intestinal epithelial cell dysfunction and ERK/MAPK signaling pathway. In addition, Luo et al. (2023) reported that HBM-EXOs are highly involved in the glucose metabolism of infants by reshaping gut microbiota toward favorable to the growth of bifidobacteria.

### 3.3 Limitations of milk-derived exosomes

Nonetheless of eminent potential in therapeutic and cosmetic field, the separation of exosomes from milk has remained as a hurdle in applying milk-derived exosomes so far. As milk contains multiple bioactive components, such as milk fat globules, casein micelles, and lipids, the separation of exosomes is essential to precisely elucidate the properties and function of milk-derived exosomes (Jukkola and Rojas, 2017). However, it is often considered as a significant challenge since milk fat globules, and casein micelles are unable to be separated to exosomes since their size are overlapped with exosomes (Blans et al., 2017; Hu Y. et al., 2021). On the other hand, contamination is other important consideration to preparation since raw milk mostly contains microorganisms, requiring a sterilization. However, sterilization at high temperature can results in quantitative and qualitative loss of exosomes. Furthermore, a recent study pointed that pasteurization, treating low temperatures, has also affected the quality of exosomes, such as membrane integrity and cargo of exosomes, while it barely influenced quantitatively (Kleinjan et al., 2021). Therefore, a novel strategy to separate exosomes from milk is required for their practical applications.

## 4 Microbial membrane vesicles

Microorganisms are in symbiosis with the host, and their metabolites are substantially associated to host health by affecting host homeostasis and regulating immune function. Thus, the influence of microorganisms and their extracellular vesicles have been intensively studied in medicinal fields. Particularly, probiotics may have health benefits when consumed or applied to the body. And they are widely believed to have excellent efficacy on treating gastrointestinal dysbiosis, systemic metabolic diseases, and from genetic disorders to complex neurodegenerative diseases so far (Aponte et al., 2020). Recently, *Lactobacillus* and *Bifidobacterium* species have been frequently used as probiotics (Williams, 2010). Theoretically, prokaryotes do not have an endoplasmic reticulum, thus, they cannot produce exosomes. However, they express exosome-like vesicles, having a membrane structure, and these play a key role in paracrine signaling between microorganisms. In recent years, mounting evidence revealed the existence of these nanovesicles and termed these to membrane vesicles. Interestingly, according to the recent studies, the membrane vesicles, released from microbes, are reported to play a critical role in physiology and immunology of the host (Deatherage and Cookson, 2012; Kaparakis-Liaskos and Ferrero, 2015; Vargoorani et al., 2020). Furthermore, the host-bacteria interaction is found to be mediated by various biomolecules that reflect the cellular origin of the bacterial derived membrane vesicles (BMVs) (Jahromi and Fuhrmann, 2021). Typically, BMVs are classified to two categories; outer membrane vesicles (OMVs) and inner membrane vesicles (IMVs), and these two groups have distinct features since their membrane composition are quite different. BMVs contain various biologically active molecules in similar exosomes and each components have a distinct physiological function. For example, their proteins are involved in stimulating the host immune system, but lipids affect membrane dynamics and interactions with host cells. On the other hand, nucleic acids, including DNA, mRNA, and non-coding RNA, can regulate gene expression in recipient cells (Hosseini-Giv et al., 2022). Additionally, BMVs are stable under physiological conditions, and their surfaces can be engineered with the genetic manipulation. Due to these favorable properties, BMVs have been spotlighted as promising candidates for the treatment of diseases, including anti-inflammation, tumors, anti-aging, and resistant infections (Jahromi and Fuhrmann, 2021).

### 4.1 *Lactobacillus*-derived membrane vesicles

*Lactobacilli* are typical probiotic Gram-positive bacteria that produce spherical EVs in the culture medium, ranging in size from 20 to 200 nm (Kurata et al., 2022). Interestingly, *Lactobacillus*-derived EVs (LEVs), which carry metabolic intermediates, proteins, and RNAs as cargo, have been reported to function as modulators of immunity and antibacterial agents. In particular, the immunoregulatory properties of LEVs have been actively studied in the fields of intestinal and skin inflammation and liver disease. Among *Lactobacillus* species, *L. plantarum* has been used for decades to enhance human intestinal mucosal immunity and improve skin barrier integrity, due to their due to their immunostimulatory and antioxidant effects (Shin et al., 2016; Kim et al., 2018). In this context, studies on the therapeutic effects of *Lactobacillus plantarum*-derived EVs (LpEVs) on intestinal and skin barriers have been reported. Kurata et al. (2022) investigated the immunostimulatory effects of LpEVs isolated from the intestinal tract of mice. In this study, they suggested that LpEVs induce immunostimulatory effects, including innate and acquired immune cell responses, through TLR2-mediated NF-κB activation. In the meantime, Kim et al. (2020) investigated the effect of LpEVs on skin immunity, especially macrophage polarization. They confirmed that LpEVs induce secretion of the anti-inflammatory cytokine IL-10 and the immunomodulatory cytokines IL-1β and GM-CSF in human skin organ culture. Based on these results, they claimed that LpEVs can improve the phenotype of highly inflammatory skin conditions and inflammatory skin diseases by correcting the macrophage imbalance (Kim et al., 2020). Meanwhile, Jo et al. (2022) noted the skin anti-aging effect of LpEVs. They demonstrated that LpEVs increase cell proliferation in skin fibroblasts and regulate mRNA expression of ECM-associated genes. In addition, they exhibited inhibition of wrinkle formation and pigmentation in clinical trials. With this regard, they suggest that LpEVs can be applied as effective anti-aging agents and effective anti-pigmentation agents to improve skin aging (Jo et al., 2022).

On the other hand, *Lactobacillus paracasei*-derived EVs (LcEVs) have been intensively investigated as an anti-inflammatory therapeutic (Pan et al., 2014; D’Auria et al., 2021). According to the report of Choi *et al.*, oral administration of LcEVs protects against DSS-induced colitis, reducing weight loss, maintaining colon length, and reducing disease activity index (DAI). They also demonstrated that LcEVs alleviate LPS-induced inflammation in the intestine by activating ER stress (Choi et al., 2020). The results of this study led to the investigation of further therapeutic effects of LcEVs. Lee *et al.* investigated the TNF-α induced skin inflammation phenotypic improvement potential of LcEVs. As a result, they confirmed that LcEVs can be efficiently absorbed into human skin cells and significantly improve TNF-a-induced skin disorders and reduced collagen synthesis. Similarly, the anti-inflammatory effect of *Lactobacillus* rhamnosus GG-derived EVs (LGEVs) has also been investigated. For instance, Tong et al. (2021b) showed that LGEVs improve intestinal inflammation by inhibiting TLR4-NF-κB-NLRP3 axis activation and altering metabolic pathways in the gut microbial community. Moreover, Gu et al. (2021) reported the barrier-enhancing function of LGEVs. They demonstrated that LDNPs protect the intestinal barrier by increasing tight junction protein expression in epithelial cells and protecting against LPS-induced inflammatory responses in macrophages. They also argued that enhancing the barrier function of LGEVs inhibited bacterial translocation and endotoxin release in alcohol-associated liver disease (ALD) mice (Gu et al., 2021). In conclusion, mounting studies on the anti-inflammatory effect of LEVs suggest that EVs derived from various *lactobacillus* species have the potential for attractive therapeutic agents to improve inflammatory bowel, skin, and liver diseases.

### 4.2 *Bifidobacterium*-derived membrane vesicles

*Bifidobacterium* strains are symbiotic microorganisms commonly found in the human gut and are known to interact with immune cells (O'Mahony et al., 2010). In early 2010s, Lopez et al. (2011) reported that exposure of dendritic cells (DCs) to *Bifidobacterium bifidum in vitro* induces the polarization of naive T cells into functional Treg cells. And through further study, they demonstrated that the ability of DC to strongly promote the differentiation ability of Treg cells could also be induced by *B. bifidum* membrane vesicles (Lopez et al., 2012). Based on these results, studies on the therapeutic functions of Bifidobacterium-derived EVs (BEVs) have gained attention, especially in immunomodulatory properties. For example, Kim et al. (2016) investigated the food allergy-alleviating effects of *Bifidobacterium longum* EVs (BlEVs). They reported that BlEVs specifically induce apoptosis of mast cells without affecting the T-cell immune response, thus alleviating food allergy symptoms (Kim et al., 2016). Furthermore, Mandelbaum et al. (2023) characterized the size, protein content, and immunomodulatory effects of BlEVs. They observed that BlEVs, which are 150 nm in size, are rich in ABC transporters and quorum sensing proteins that contribute to the regulatory effect on the host immune system. In conclusion, they suggested that the anti-inflammatory effect of BlEVs, inducing IL-10 secretion in immune cell coculture, was due to the internal protein of BlEVs (Mandelbaum et al., 2023). Various probiotic-derived BEVs contribute to a clearer understanding of the mechanisms involved in probiotic properties, while also opening opportunities for the development of novel immunomodulatory treatments.

### 4.3 *Saccharomyces*-derived exosomes

*Saccharomyces cerevisiae* (*S. cerevisiae*), also known as bread yeast, has been reported to regulate the host immune response, such as enhancing the innate immune response and maturation immune cells (Chou et al., 2017; Bazan et al., 2018). Given that EVs reflect the properties of donor cells, the characteristics and functions of EVs isolated from *Saccharomyces* (SEVs) have recently been investigated. Higuchi et al. (2023) defined the physical properties and biological functions of *S. cerevisiae* EVs (ScEVs). In their research, they found that ScEVs contain β-D-glucans, a bioactive component with anti-cancer, anti-inflammatory, and immunomodulatory properties. Furthermore, they reported that ScEVs are taken up by immune cells via endocytosis and that the interaction between TLR2 and the cargo of ScEVs activates the immune response of macrophages and dendritic cells (Thomas et al., 2022; Higuchi et al., 2023). Meanwhile, Mierzejewska et al. (2023) focused the characteristics that SEVs can be internalized into cells *via* endocytosis. In their study, they loaded the SEVs with the anticancer drug doxorubicin, added it to the cell culture, and evaluated its cytotoxicity. As a result, they confirmed the activity of doxorubicin, suggesting its availability as a drug carrier. In addition, they expected that genetic modification of yeast would allow modification of the surface protein composition of SEVs. Taking all of the above into account, they suggested SEVs as promising drug delivery candidates (Mierzejewska et al., 2023).

### 4.4 Limitations of microbial membrane vesicles

Although they have demonstrated clear therapeutic potential at the preclinical level, BMVs and SEVs have not yet been fully adopted into clinical practice. Due to their microbially derived nature, BMVs and SEVs pose a number of challenges. For example, the metabolic turnover of microorganisms during culture affects the structural features of BMVs and SEVs and the composition of their internal contents. In addition, impurities in the culture must be purified, as various substances derived from microorganisms can cause sepsis and other inflammatory diseases. Furthermore, since no standards have been developed to evaluate the properties of vesicles, comparative evaluation of vesicles produced under different conditions or holistic analysis through metastudies is needed.

## 5 Discussions and future prospects

Due to their therapeutic potential at low production costs, various alternatives of MSC-EXOs have recently attracted attention. With considerable effort, they have been actively commercialized as active ingredients in cosmetics or food supplements. However, their therapeutic applications have been limited, since the precise isolation of exosomes or exosome-like nanovesicles has remained a significant challenge so far. Unlike cells cultured *in vitro*, raw plant-derived nanovesicles contain a variety of components and fragmented tissues, which make accurate separation difficult using conventional size-based purification methods. In addition, the high fat content of milk, together with the presence of various proteins such as immunoglobulins and caseins and their aggregates, make accurate separation of exosomes difficult. Similarly, in the case of membrane structures derived from microbes, the various components are a potential risk factor for potentially lethal side effects. As most of these alternatives have not been studied for biomarkers as much as mammalian cell-derived exosomes, there are currently no clear purification methods or methods for the evaluation of purified exosomes and exosome-like nanovesicles, and this uncertainty greatly limits their use as therapeutics. In addition, comparisons with other types of exosomes or exosome-like nanovesicles remain unclear and unbalanced because the active ingredient in each vesicle is different and most comparisons are based on case-by-case risk-benefit assessments. On the other hand, consideration should be given to formulation or engineering modifications to improve the stability of exosomes or exosome-like nanovesicles and to ensure that they retain active ingredients (Nguyen et al., 2022). Therefore, further research on the biogenesis, biomarkers, and bioactive ingredients of each exosome or exosome-like nanovesicles is needed to develop accurate isolation and characterization methods.
